# Taurine Protects against Silica Nanoparticle-Induced Apoptosis and Inflammatory Response via Inhibition of Oxidative Stress in Porcine Ovarian Granulosa Cells

**DOI:** 10.3390/ani14202959

**Published:** 2024-10-14

**Authors:** Fenglei Chen, Jiarong Sun, Rongrong Ye, Tuba Latif Virk, Qi Liu, Yuguo Yuan, Xianyu Xu

**Affiliations:** 1 College of Veterinary Medicine, Yangzhou University, Yangzhou 225009, China; 15996825403@163.com (J.S.); yrr971106@163.com (R.Y.); xeroxhunt@gmail.com (T.L.V.); qiliu@yzu.edu.cn (Q.L.); yyg9776430@163.com (Y.Y.); 2 Jiangsu Co-Innovation Center for Prevention and Control of Important Animal Infectious Diseases and Zoonoses, Yangzhou 225009, China; 3 Joint International Research Laboratory of Agriculture and Agri-Product Safety of the Ministry of Education of China, Yangzhou University, Yangzhou 225009, China

**Keywords:** taurine, silica nanoparticles, apoptosis, oxidative stress, granulosa cells

## Abstract

**Simple Summary:**

Taurine (Tau) is a well-known antioxidant. However, the protective effects of Tau against silica nanoparticle (SNP)-induced reproductive toxicity still remain unexplored. So this study reveals the effect of Tau on SNP-induced porcine ovarian granulosa cell toxicity. SNPs trigger oxidative stress through excessive ROS production, leading to inflammation and cell apoptosis in porcine ovarian granulosa cells. Tau mitigates SNP-induced cytotoxicity by reducing oxidative stress, inflammatory response, and mitochondria-mediated cell apoptosis in porcine ovarian granulosa cells. Therefore, Tau could be an effective strategy to alleviate SNP-induced toxicity and holds promising application prospects in the animal husbandry and veterinary industry.

**Abstract:**

Silica nanoparticles (SNPs) induce reproductive toxicity through ROS production, which significantly limits their application. The protective effects of taurine (Tau) against SNP-induced reproductive toxicity remain unexplored. So this study aims to investigate the impact of Tau on SNP-induced porcine ovarian granulosa cell toxicity. In vitro, granulosa cells were exposed to SNPs combined with Tau. The localization of SNPs was determined by TEM. Cell viability was examined by CCK-8 assay. ROS levels were measured by CLSM and FCM. SOD and CAT levels were evaluated using ELISA and qPCR. Cell apoptosis was detected by FCM, and pro-inflammatory cytokine transcription levels were measured by qPCR. The results showed that SNPs significantly decreased cell viability, while increased cell apoptosis and ROS levels. Moreover, SOD and CAT were decreased, while IFN-α, IFN-β, IL-1β, and IL-6 were increased after SNP exposures. Tau significantly decreased intracellular ROS, while it increased SOD and CAT compared to SNPs alone. Additionally, Tau exhibited anti-inflammatory effects and inhibited cell apoptosis. On the whole, these findings suggest that Tau mitigates SNP-induced cytotoxicity by reducing oxidative stress, inflammatory response, and cell apoptosis. Tau may be an effective strategy to alleviate SNP-induced toxicity and holds promising application prospects in the animal husbandry and veterinary industry.

## 1. Introduction

With the advancements in nanotechnology, nano-materials, such as silica nanoparticles (SNPs), have rapidly developed in animal husbandry and veterinary industry. Due to their mature synthesis process, good stability, favorable surface modification, unique hollow structure, large specific surface area, and strong adsorption capacity, SNPs are widely used as carriers in feed additives [1], veterinary drugs [2], and animal vaccines [3,4,5]. In terms of feed additives, SNPs effectively improve feed utilization, increase the weight of piglets, and reduce the mortality and diarrhea rate of piglets after weaning [6,7]. In the field of veterinary drugs, SNPs are ideal for preparing sustained-release drugs and improving the utilization rate of insoluble drugs. SNP-loaded drugs offer advantages such as effective slow-release, high utilization, reduced dosage, minimal residue, and nontoxic in pigs [8,9]. In the aspect of vaccine research, SNPs mainly focus on the antigen-carrier system and adjuvant effect, offering better immune responses and lower toxicity compared to traditional adjuvants. SNPs have been widely used in the development of vaccines against porcine circovirus, foot-and-mouth disease virus, and more [10,11].

SNPs can cause reversible damage after withdrawal at low doses [12]. Furthermore, SNPs have been shown to enhance wound healing ability and slow down aging through potential hermetic effects [13]. In addition, previous studies have also demonstrated that low doses of SNPs can enhance testosterone secretion in mouse Leydig cells [14]. However, high doses of SNPs may have harmful effects. Raziye Mohammadpour et al. found that the mice at their 10-day maximum tolerated dose need up to one year to recover from the acute toxic effects of SNPs [15]. The toxicity of SNPs largely depends on their physicochemical properties. Due to their small particle size, SNPs are able to penetrate physiological barriers, enter the blood circulation to reach various organs, and accumulate in non-target tissues, leading to acute and chronic toxicity [16]. The ovary possesses a unique network of small blood vessels and lacks a protective barrier similar to the blood–testis barrier in the male reproductive system and is particularly susceptible to SNP accumulation [17]. This can result in follicular atresia, induce reproductive toxicity, and reduce animal fecundity in animals [18,19]. Due to the large specific surface area, SNPs exhibit excellent redox reaction activity, and produce excessive reactive oxygen species (ROS), which leads to ROS-mediated oxidative stress and endoplasmic reticulum stress, resulting in cell apoptosis and inflammatory responses [20]. In terms of reproductive system toxicity, in vivo studies have shown that SNPs induce ovarian granulosa cell apoptosis and follicular atresia through activation of oxidative stress [18]. Similarly, in vitro studies have shown that SNPs induce ovarian granulosa cell apoptosis and follicular atresia via induction of oxidative stress [21].

Taurine (2-aminoethanesulfonic acid, Tau) is an endogenous sulfonic amino acid that can also be obtained from dietary sources and pharmaceutical supplements to improve the growth of pig [22,23]. Besides improving meat quality, muscle fiber type, and mitochondrial function, Tau is essential for the development of the central nervous system and reproductive system [24,25]. Maria V.T. Lobo et al. report that Tau increases the number of primary and antral follicles, while reducing follicular atresia in the ovary [26]. It is well known that Tau is regarded as a cytoprotective molecule with antioxidant activity, eliminates anti-inflammatory properties, and prevents calcium accumulation [27]. Previous studies have shown that SNPs induce ovarian granulosa cell apoptosis, leading to ovarian toxicity via oxidative stress [18,21]. Due to the antioxidant efficacy of Tau, this study aims to determine whether Tau can mitigate SNP-induced cytotoxicity in porcine ovarian granulosa cells. In the current study, porcine ovarian granulosa cells were cultured in vitro and the cytotoxicity of SNP exposure was examined. Thereafter, the effects of exogenous Tau administration on SNP-induced cell cytotoxicity were then explored. We hope that Tau administration can improve biosafety and strengthen the clinical application of SNPs.

## 2. Materials and Methods

### 2.1. Porcine Ovarian Granulosa Cell Isolation and Culture In Vitro

Porcine ovaries were obtained from healthy sows at a local standardized slaughterhouse. The normal ovaries were placed in sterile 37 °C saline containing 100 U/mL penicillin and 100 μg/mL streptomycin, and rapidly delivered to the laboratory within 2 h. In the laboratory, the collected ovaries were washed three times each with 75% ethanol and normal saline, and then placed in saline at a constant temperature of 37 °C. To collect porcine ovarian granulosa cells, follicular fluid from luminal follicles (3–6 mm in diameter) was collected using a 26-gauge needle. The follicular fluid was added with PBS (pH 7.4) and filtered through a 400-mesh filter to eliminate oocytes. The mixture was centrifuged at 110× *g* for 3 min. The trypan blue exclusion test was used to determine the viability of porcine ovarian granulosa cells. 2 × 10^6^ cells/4 mL/well were re-suspended in D/F-12 medium supplemented with 10% fetal bovine serum (FBS), seeded into 60 mm culture dishes, and cultured at 37 °C under a 5% CO_2_ atmosphere. FSHR immunohistochemistry was used to detect the purity of porcine ovarian granulosa cells.

### 2.2. Silica Nanoparticle Preparation and Cell Treatment

SNPs were prepared and synthesized by the Stöber method as described previously [24]. The particle morphology was measured by transmission electron microscopy (TEM, Tecnai 12, Royal Philips, Amsterdam, The Netherlands). Image J software (Version 1.8.0, National Institutes of Health, Bethesda, MD, USA) was used to analyze the average particle diameter of SNPs. The hydrodynamic size and zeta potential of SNPs were determined by a Zetasizer (3000HS, Malvern Instruments Ltd., Worcestershire, UK).

To reveal the effects of SNP-induced cytotoxicity, lethal doses of SNPs were used in porcine ovarian granulosa cells in vitro. Based on the previous reports, an estimated SNP intake through oral administration is up to 1.8 mg/kg bw/day [28,29], the corresponding in vitro exposure doses of SNPs in a 96-well plate per well are 0 to 262.8 μg/mL, which is equivalent to the doses of a 50 kg person exposed to SNPs daily over a 5-year time period [30,31]. Porcine ovarian granulosa cells were seeded in 60 mm culture dishes. When the cell confluence reached 70 to 80%, the cells were treated with different doses of SNPs (100, 200, 300, 400, 500, 600, 800, and 1000 μg/mL) for 48 h, and then the cells were collected for the subsequent experiments. To further explore the effect of Tau on SNP-induced cytotoxicity, 10 mM taurine was treated with porcine ovarian granulosa cells for 48 h to choose an optimum dose, and subsequently combined with SNPs for further study.

### 2.3. Determination of SNP Localization

Porcine ovarian granulosa cells were exposed to SNPs at the dose of 200 μg/mL for 24 h. The cells were washed three times with PBS (pH 7.4) to remove the excess SNPs, collected after trypsinization, and re-suspend in PBS (pH 7.4) containing 2.5% glutaraldehyde and 4% paraformaldehyde for 3 h. After washing with PBS, the cells were placed in 2% agarose gel and subsequently fixed with 4% osmic acid for 1 h. Following a wash with distilled water, the cells were stained with uranium dioxyacetate for 1 h. Then, the cells were dehydrated through a graded ethanol series and embedded with epoxy resin. Ultrathin slices were prepared and stained with 5% uranium dioxyacetate and 2% lead citrate for TEM (HT7800, Hitachi, Tokyo, Japan).

### 2.4. Cell Viability Assay

The CCK-8 method was used to evaluate the effects of SNPs and Tau on cell viability of porcine ovarian granulosa cells. Briefly, the cells were seeded in 96-well plates at a density of 1 × 10^4^ cells/200 μL/well and cultured for 24 h. Different doses of SNPs (100, 200, 300, 400, 500, 600, 800, and 1000 μg/mL) diluted with D/F-12 medium were exposed to the cells for 48 h, using D/F-12 medium alone as a control group. Subsequently, 10 μL of CCK-8 was added to each well and incubated for 2 h at 37 °C under a 5% CO_2_ atmosphere. After incubation, OD values were measured at a wavelength of 450 nm using a microplate reader (Model 680, Bio-Rad, Hercules, CA, USA).

### 2.5. Determination of Lactate Dehydrogenase (LDH) Level

The LDH assay was used to evaluate the effects of SNPs and Tau on the cytotoxicity of porcine ovarian granulosa cells. The cells were seeded in 96-well plates at a cell density of 1 × 10^4^ cells/200 μL/well. Then, different doses of SNPs (100, 200, 300, 400, 500, 600, 800, and 1000 μg/mL) were exposed to the cells for 24 h. The medium was collected and added to other 96-well culture dishes. Subsequently, 150 μL LDH was added to each well and continually incubated for 2 h. After incubation, 120 μL supernatant was taken from each well and added to a new 96-well plate for determination. OD values were measured at a wavelength of 490 nm using a microplate reader (Model 680, Bio-Rad, Hercules, CA, USA).

### 2.6. Measurement of ROS Production

Dichlorodihydrofluorescein diacetate (DCFH-DA) was used to measure intracellular ROS generation in porcine ovarian granulosa cells after SNP and Tau exposures. The cells were incubated in 1 mL DCFH-DA (10 μM) for 30 min at 37 °C in the dark. After removing the DCFH-DA solution, the cells were then washed with PBS (pH 7.4). The cells were stained with Hoechst (Beyotime, Shanghai, China), and subsequently, the fluorescent images of the cells were captured by confocal laser scanning microscopy (CLSM, TCS SP8 STED, Leica, Wetzlar, Hessen, Germany). Additionally, the cells were used to measure the fluorescence intensity by flow cytometry (FCM, Beckman Coulter Cytomics Altra, Brea, CA, USA).

### 2.7. Determination of Superoxide Dismutase (SOD) and Catalase (CAT) Enzyme Activities

Porcine ovarian granulosa cells were treated with SNPs and Tau and collected into centrifuge tubes, and the supernatant was discarded after centrifugation. The cells were lysed by ultrasound after the extraction solution was added in a ratio of 500:1. The mixture was centrifuged at 12,000× *g* at 4 °C for 10 min. The supernatant was transferred to new tubes and placed on ice until testing. The samples were analyzed using commercial kits (Beijing Solarbio Science & Technology, Beijing, China). The experimental protocol was carried out according to the instructions by enzyme-linked immunosorbent assay (ELISA). Each sample was tested in triplicate.

### 2.8. FCM Analysis

FCM was used to determine cell apoptosis in porcine ovarian granulosa cells. According to the instructions of an apoptosis detection kit (KeyGen Biotech, Nanjing, China), ovarian granulosa cells were digested with trypsin after SNP exposures. The cells were then collected after centrifugation and re-suspended in a staining buffer containing Annexin V-FITC and PI. Finally, FCM (Beckman Coulter Cytomics Altra, Brea, CA, USA) was performed to measure the cell apoptotic rate within 1 h.

### 2.9. RNA Extraction and Quantitative Polymerase Chain Reaction (qPCR)

Total RNA was extracted from porcine ovarian granulosa cells using TRIzol kits (TaKaRa Bio, Inc., Dalian, China). The cDNA was synthesized using a reverse transcription reagents kit (TaKaRa Bio, Inc., Dalian, China). qPCR amplification was performed using the SYBER green probe (TaKaRa Bio, Inc., Dalian, China), to calculate the relative quantification of the gene levels. Each sample was tested in triplicate, and relative mRNA levels were normalized to the expression levels of the housekeeping gene *GAPDH*. The primers used are described in Table 1. 

### 2.10. Western Blot

Porcine ovarian granulosa cells were treated with radio-immunoprecipitation assay buffer (RIPA) containing 1% phosphatase inhibitor and 1% protease inhibitor cocktail (KeyGen Biotech, Nanjing, China) to extract total cell protein. The lysate was centrifuged at 12,000× *g* at 4 °C for 15 min to obtain the protein. The protein concentration was determined using the BCA protein assay kit (KeyGen Biotech, Nanjing, China). Total protein (20 μg) was then separated by 12% sodium dodecyl sulfate-polyacrylamide gel electrophoresis (SDS-PAGE). The proteins of interest on the gel were transferred onto polyvinylidene difluoride (PVDF) membranes via electroblotting (Millipore, Bedford, MA, USA). The membranes were blocked with 5% skim milk, followed by overnight incubation at 4 °C with antibodies cleaved PARP (T55035; 1:1000 dilution, Abmart, Shanghai, China), cleaved caspase-3 (9664; 1:1000 dilution, Cell Signaling Technology (CST), Danvers, MA, USA), BCL-2 (ab182858; 1:1000, Abcam, Cambridge, MA, USA), BAX (ab32503; 1:1000, Abcam, Cambridge, MA, USA), and β-actin (AC026; 1:2000, ABclonal, Wuhan, China). After washing with Tris-buffered saline containing Tween 20 (TBST), the membranes were incubated with a secondary antibody conjugated with horseradish peroxidase (HRP, 7074; 1:5000, CST) for 1 h at room temperature. Immunoreactive bands were then visualized using an ultrasensitive ECL chemiluminesence detection kit (Thermo Fisher Scientific, Waltham, MA, USA) and a gel imaging system (Tannon Science & Technology, Shanghai, China). The strip density was analyzed with Quantity One software (Version 4.6.2, Bio-Rad, Hercules, CA, USA). Experiments were performed independently in triplicate.

### 2.11. Statistical Analysis

All data are presented as mean ± standard error of the means (SEM). Each experiment was performed in triplicate and repeated three times. Statistical analysis was conducted using Statistical Package for the Social Sciences (SPSS) software (Version 18.0, Chicago, IL, USA). Statistical significance of the differences was analyzed by one-way ANOVA and comparisons between the control and treated groups were performed by LSD test. Statistical significance was shown at * *p* < 0.05.

## 3. Results

### 3.1. Effect of SNPs on Cell Viability and Apoptosis

**Figure 1:**
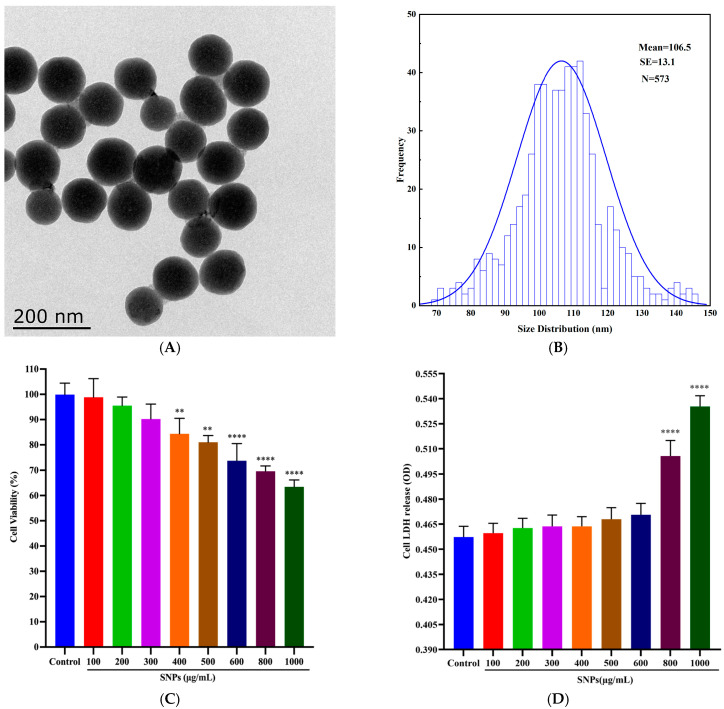
Characteristics and cytotoxicity of SNPs: (**A**) Representative TEM image of SNPs. (**B**) Size distribution of SNPs. (**C**) CCK-8 assay. (**D**) LDH leakage assay. ** *p* < 0.01, and **** *p* < 0.0001 vs. Control.

TEM results showed that SNPs had a spherical shape (Figure 1A) with an average diameter of about 106.5 ± 13.1 nm (Figure 1B). The average hydrodynamic size was 110.8 ± 26.5 nm in physiological saline solution; the zeta potential was −49.7 ± 26.5 in physiological saline solution, as described in our previous study [14].

Porcine ovarian granulosa cells were cultured in vitro and the purity was determined by FSHR staining (Figure S1). To examine the cytotoxicity of SNPs on porcine ovarian granulosa cells, the cells were obtained from porcine ovaries and cultured in vitro. CCK-8 results showed that cell viability did not make a significant difference for 48 h after 100 to 300 μg/mL SNP exposures compared to the control (Figure 1C). However, SNP exposures (above 400 μg/mL) significantly decreased the viability in a dose-dependent manner (Figure 1C). To assess the damage of ovarian granulosa cells after SNP exposures, the results of the LDH assay showed that there were no significant differences after 100 to 600 μg/mL SNP exposures compared to the control (Figure 1D). However, SNP exposures (above 800 μg/mL) significantly increased the leakage of LDH (Figure 1D).

### 3.2. Cellular Internalization of SNPs in Porcine Ovarian Granulosa Cells

**Figure 2:**
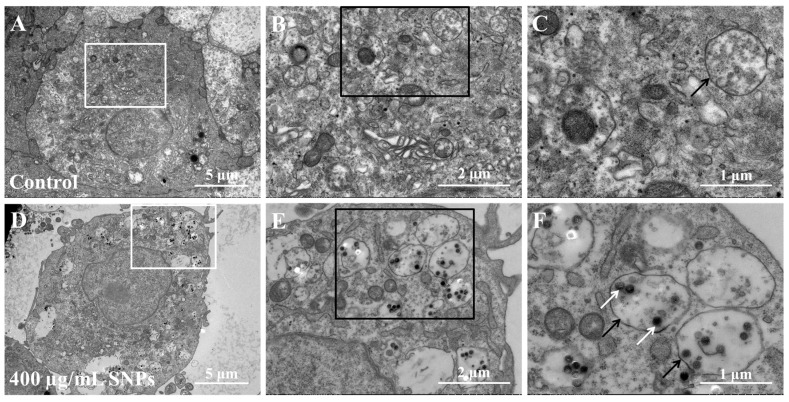
Cellular uptake and distribution of SNPs in porcine ovarian granulosa cells: (**A**) Representative TEM image in the control group. The cells were not exposed to SNPs. (**B**) Zoomed-in image of the white box in (**A**). (**C**) Zoomed-in image of the black box in (**B**). (**D**) Representative TEM image in the SNP-exposed group. The cells were exposed to 400 μg/mL SNPs for 48 h. (**E**) Zoomed-in image of the white box in (**D**). (**F**) Zoomed-in image of the black box in (**E**). Black arrows indicate vesicles in the cytoplasm and white arrows indicate SNPs. Scale bar, 5 μm (**A**,**D**), 2 μm (**B**,**E**), and 1 μm (**C**,**F**).

To detect the cellular localization of SNPs, TEM results showed that there were no nanoparticles in the cytoplasm of porcine ovarian granulosa cells in the control group (Figure 2A–C). Comparatively, electron-dense nanoparticles were present after SNP exposures (Figure 2D,E). The particles were mainly located freely in the cytoplasm or enclosed in the monolayer membranous vesicles of the cytoplasm in porcine ovarian granulosa cells in the SNP-exposed group (Figure 2E). Moreover, there were more monolayer membranous vesicles in the SNP-exposed group than in the control group (Figure 2B,E).

### 3.3. Effect of SNPs on Oxidative Stress

**Figure 3:**
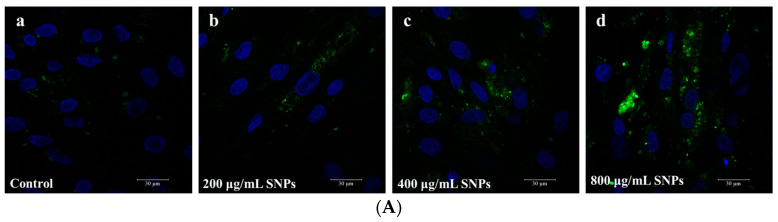

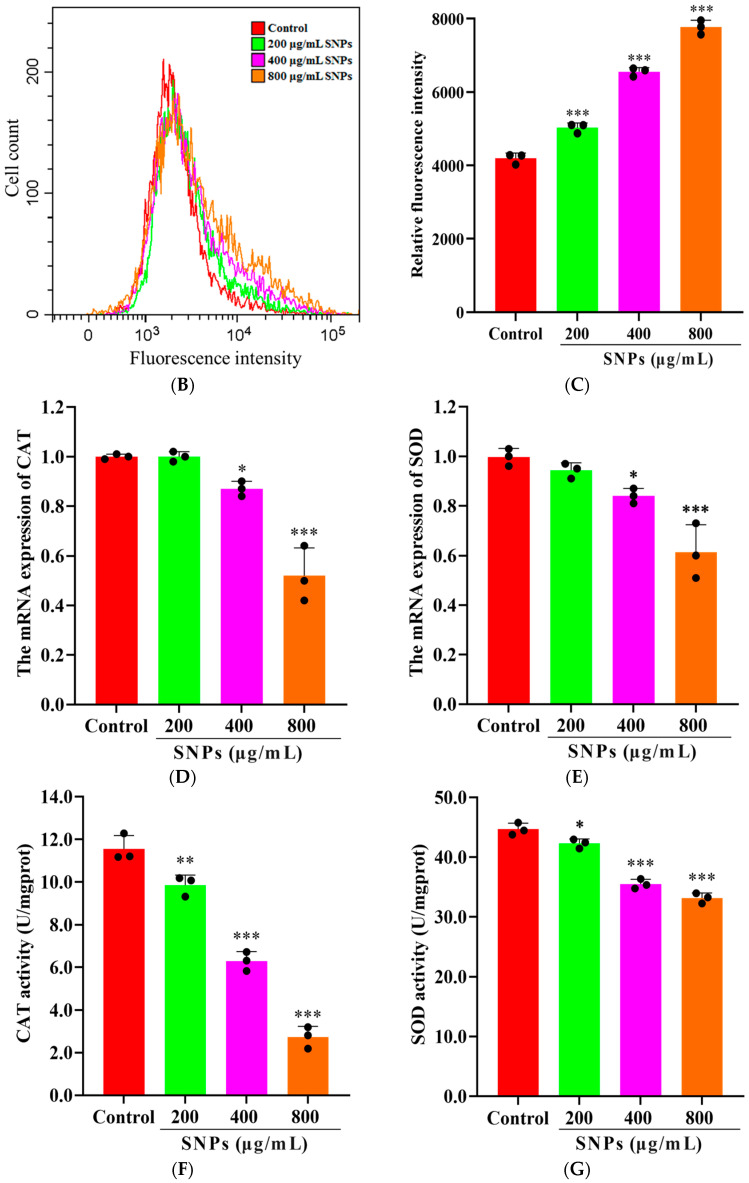
SNP-induced oxidative stress in porcine ovarian granulosa cells. (**A**) Representative images of ROS staining by CLSM. Ovarian granulosa cells were exposed to control (**a**), 200 (**b**), 400 (**c**), and 800 (**d**) μg/mL SNPs for 48 h. Green indicates fluorescence of ROS and blue indicates the nucleus of ovarian granulosa cells. Scale bar, 30 μm. (**B**) Quantitative analysis of the intracellular ROS levels by FCM. (**C**) Corresponding analysis of fluorescence intensity in Figure (**B**). (**D**) Quantitative analysis of CAT mRNA levels by qPCR. (**E**) Quantitative analysis of SOD mRNA levels by qPCR. (**F**) Quantitative analysis of CAT enzyme activity by ELISA. (**G**) Quantitative analysis of SOD enzyme activity by ELISA. * *p* < 0.05, ** *p* < 0.01, and *** *p* < 0.001 vs. Control.

To assess SNP-induced oxidative stress, intracellular ROS levels were measured by CLSM (Figure 3A) and FCM (Figure 3B,C) after SNP exposures. Clearly, CLSM and FCM results showed that the fluorescence intensities of the intracellular ROS were higher in the SNP-exposed groups compared to the control group in a dose-dependent manner (Figure 3A–C). Additionally, the mRNA levels and activities of CAT and SOD enzymes were assessed. qPCR results showed that the mRNA levels of CAT and SOD decreased in a dose-dependent manner after SNP exposures (Figure 3D,E). Similarly, ELISA results showed a decrease in the activities of CAT and SOD enzymes (Figure 3F,G).

### 3.4. Effect of SNPs on the Inflammatory Response

**Figure 4:**
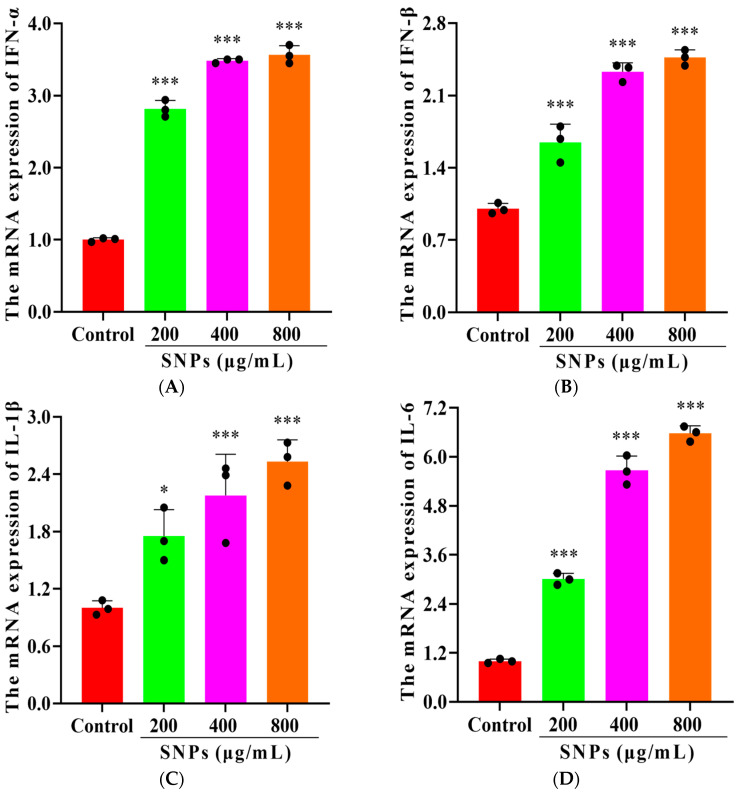
SNP-activated inflammatory response in porcine ovarian granulosa cells: (**A**–**D**) Quantitative analysis of the mRNA levels for IFN-α (**A**), IFN-β (**B**), IL-1β (**C**), and IL-6 (**D**) by qPCR. * *p* < 0.05 and *** *p* < 0.001 vs. Control.

To detect SNP-activated inflammatory response, pro-inflammatory cytokine transcriptions were determined. qPCR results showed that the mRNA levels of interferon-alpha (IFN-α), IFN-β, interleukin (IL)-1β, and IL-6 increased after 200, 400, and 800 μg/mL SNP exposures for 48 h (Figure 4).

### 3.5. Effects of SNPs on Cell Apoptosis

**Figure 5:**
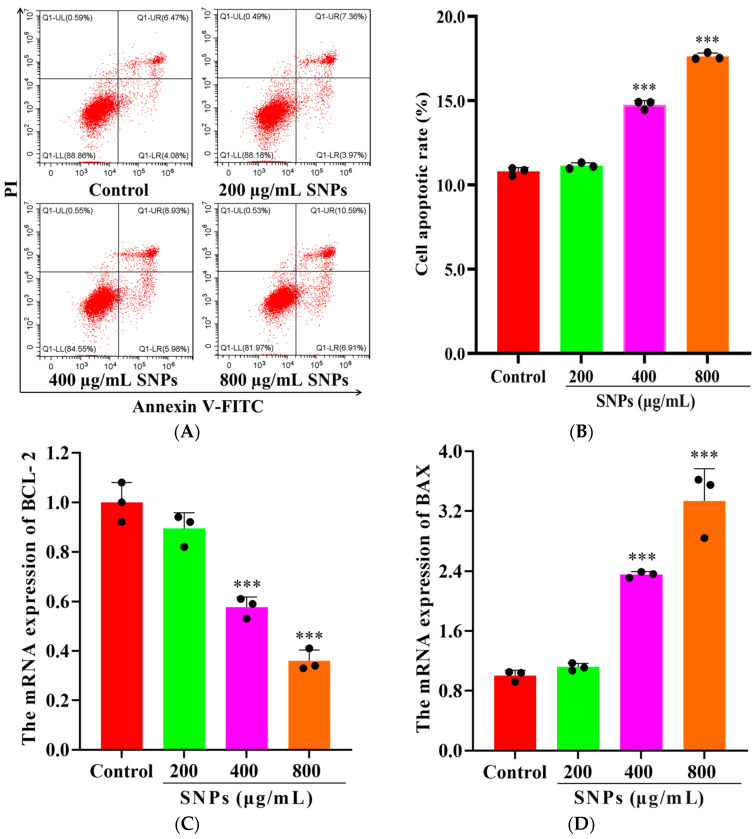

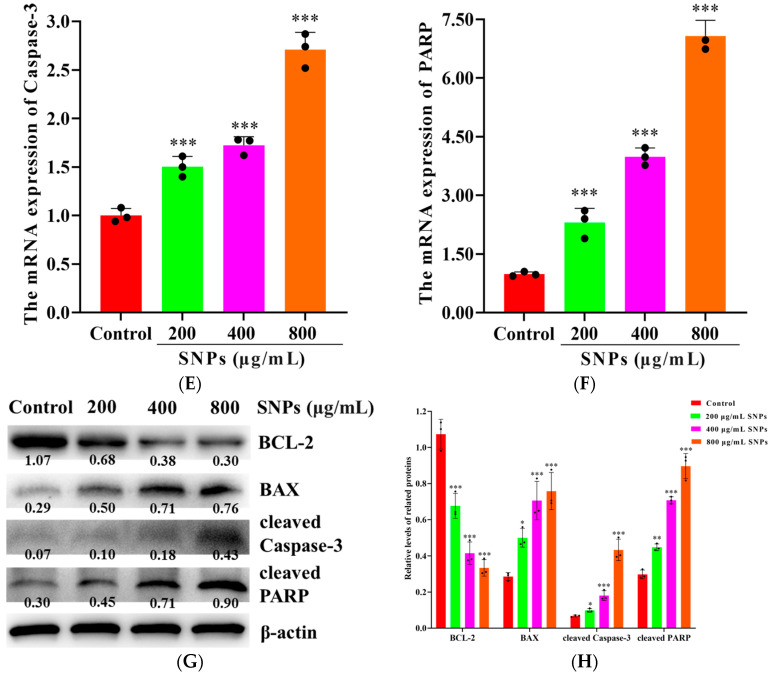
Cell apoptosis was activated in porcine ovarian granulosa cells after SNP exposures: (**A**) The apoptotic rate was determined by FCM. Q1-UL quadrant represents cell death caused by mechanical damage or necrotic cells, Q1-UR quadrant represents late apoptotic cells, Q1-LL quadrant represents the normal cells, and Q1-LR quadrant represents early apoptotic cells. (**B**) Quantification of the apoptotic rate. (**C**–**F**) Quantitative analysis of the mRNA levels for BCL-2 (**C**), BAX (**D**), Caspase-3 (**E**), and PARP (**F**) by qPCR. (**G**) Detection of BCL-2, BAX, cleaved Caspase-3, and cleaved PARP expressions by Western Blot. (**H**) Quantitative analysis of the band intensity for BCL-2, BAX, cleaved Caspase-3, and cleaved PARP. * *p* < 0.05, ** *p* < 0.01, and *** *p* < 0.001 vs. Control.

To examine SNP-induced cell apoptosis, FCM results showed apoptotic rates of (11.13 ± 0.18)%, (14.76 ± 0.26)%, and (17.63 ± 0.20)% after 48 h exposure to 200, 400, and 800 μg/mL SNPs, respectively (Figure 5A,B). SNP exposures at 400 and 800 μg/mL significantly induced cell apoptosis compared to the control (10.80 ± 0.23)%, (Figure 5A,B). qPCR results showed that the mRNA levels of BAX, Caspase-3, and PARP increased, while BCL-2 decreased after SNP exposures (Figure 5C–F). Furthermore, Western Blot results showed that the protein expressions of cleaved PARP, cleaved Caspase-3, and BAX increased, while BCL-2 decreased after SNP exposures (Figure 5G,H).

### 3.6. Effects of Tau on SNP-Induced Oxidative Stress

**Figure 6:**
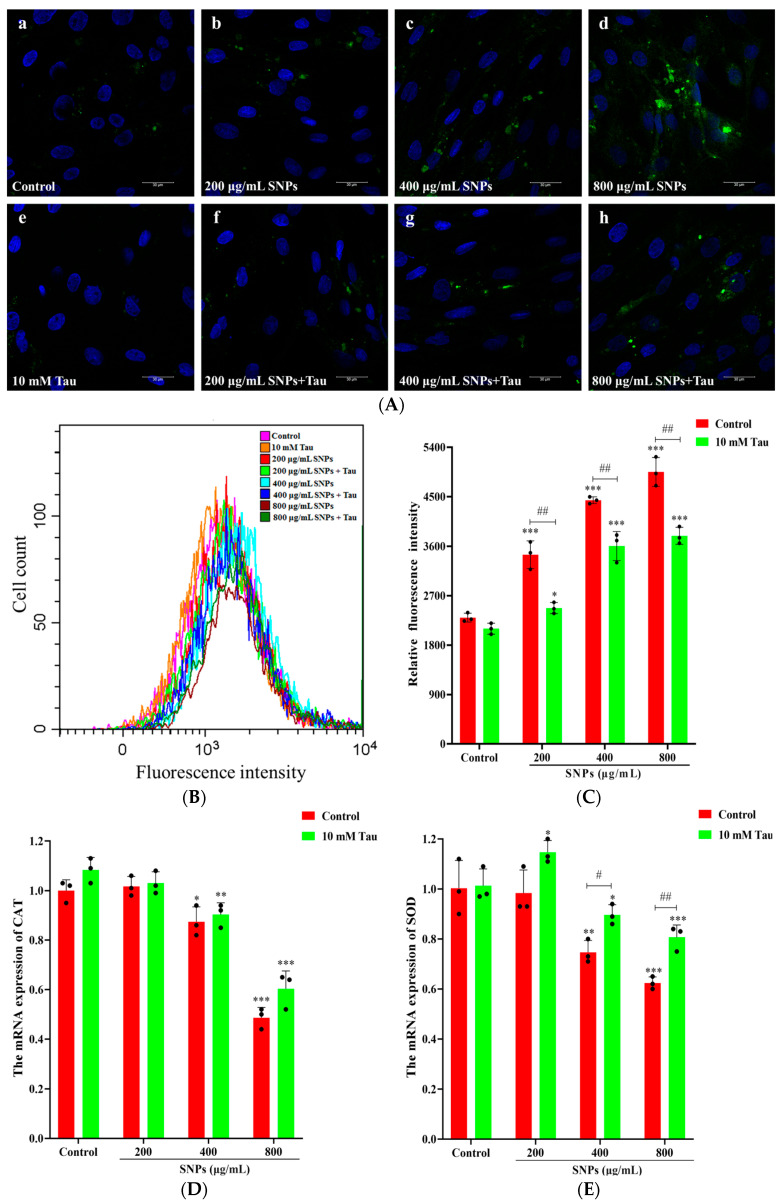

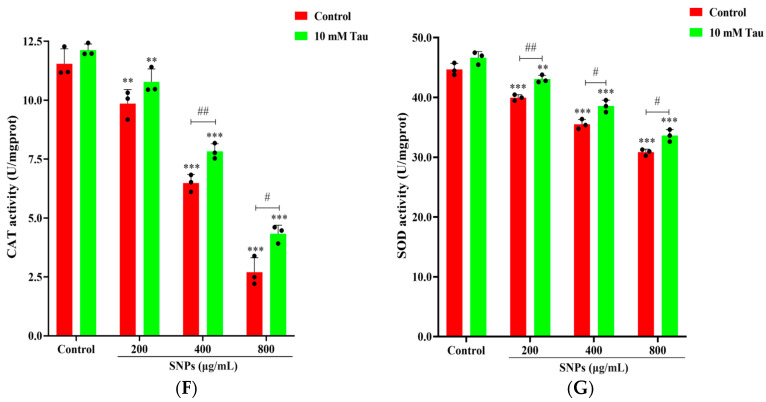
Tau inhibited SNP-induced oxidative stress in porcine ovarian granulosa cells. Ovarian granulosa cells were exposed to SNPs in the absence or presence of 10 mM Tau for 48 h: (**A**) Representative images of ROS staining by CLSM. Ovarian granulosa cells were exposed to control (**a**), 200 μg/mL SNP group (**b**), 400 μg/mL SNP (**c**), 800 μg/mL SNP (**d**), 10 mM Tau (**e**), 200 μg/mL SNP combined with 10 mM Tau (**f**), 400 μg/mL SNP combined with 10 mM Tau (**g**), and 800 μg/mL SNP combined with 10 mM Tau (**h**). Green indicates fluorescence of ROS and blue indicates the nucleus of ovarian granulosa cells. Scale bar, 30 μm. (**B**) Quantitative analysis of the intracellular ROS levels by FACS. (**C**) Corresponding analysis of the fluorescence intensity in Figure (**B**). (**D**) Quantitative analysis of CAT mRNA levels by qPCR. (**E**) Quantitative analysis of SOD mRNA levels by qPCR. (**F**) Quantitative analysis of CAT enzyme activity by ELISA. (**G**) Quantitative analysis of SOD enzyme activity by ELISA. * *p* < 0.05, ** *p* < 0.01, and *** *p* < 0.001 vs. Control. ^#^ *p* < 0.05 and ^##^ *p* < 0.01 vs. SNP-exposed group.

To explore the effects of Tau on SNP-induced oxidative stress, CLSM and FCM results showed that the fluorescence intensities of the intracellular ROS were weaker in the Tau + SNP-exposed groups compared to SNP-exposed groups (Figure 6A–C). Furthermore, qPCR results showed that the mRNA levels of SOD and CAT increased after Tau supplementation (Figure 6D,E). Similarly, ELISA results showed that the activities of SOD and CAT enzymes also increased after Tau supplementation (Figure 6F,G).

### 3.7. Effects of Tau on SNP-Induced Inflammatory Response

**Figure 7:**
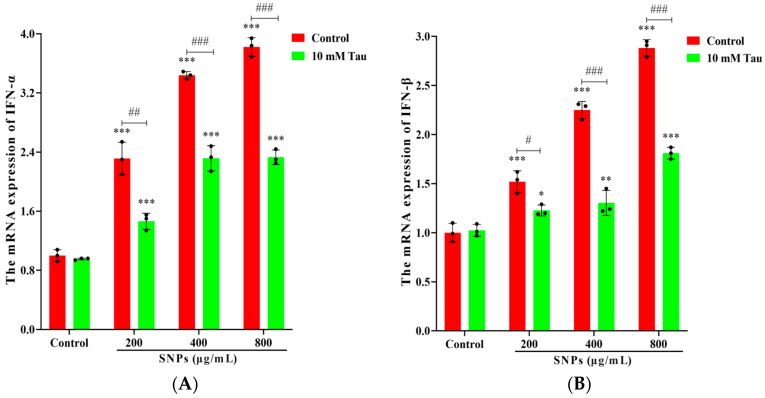

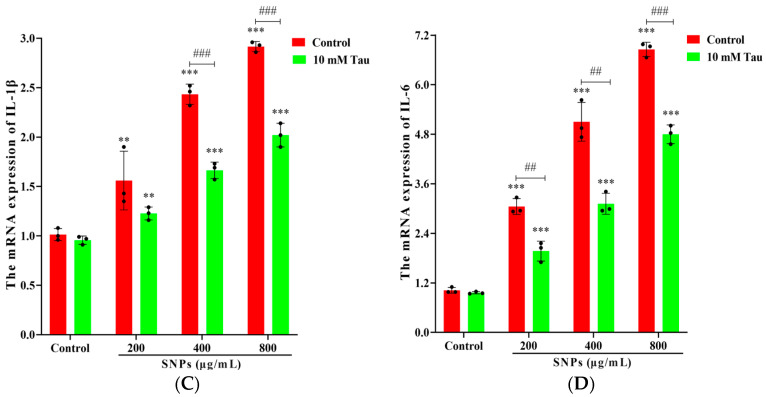
Tau inhibited SNP-activated inflammatory response in porcine ovarian granulosa cells. Ovarian granulosa cells were exposed to SNPs in the absence or presence of 10 mM Tau for 48 h: (**A**–**D**) Quantitative analysis of the mRNA levels for IFN-α (**A**), IFN-β (**B**), IL-1β (**C**), and IL-6 (**D**) by qPCR. * *p* < 0.05, ** *p* < 0.01, and *** *p* < 0.001 vs. Control. ^#^ *p* < 0.05, ^##^ *p* < 0.01, and ^###^ *p* < 0.001 vs. SNP-exposed group.

To detect the effects of Tau on SNP-activated inflammatory response, qPCR results showed that mRNA levels of IFN-α, IFN-β, IL-1β, and IL-6 decreased in the Tau + SNP-exposed groups compared to the SNP-exposed groups (Figure 7).

### 3.8. Effects of Tau on SNP-Induced Cell Apoptosis

**Figure 8:**
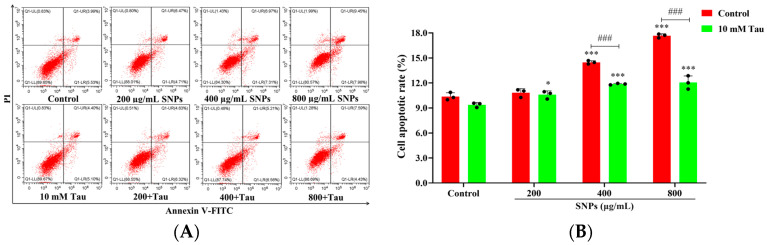

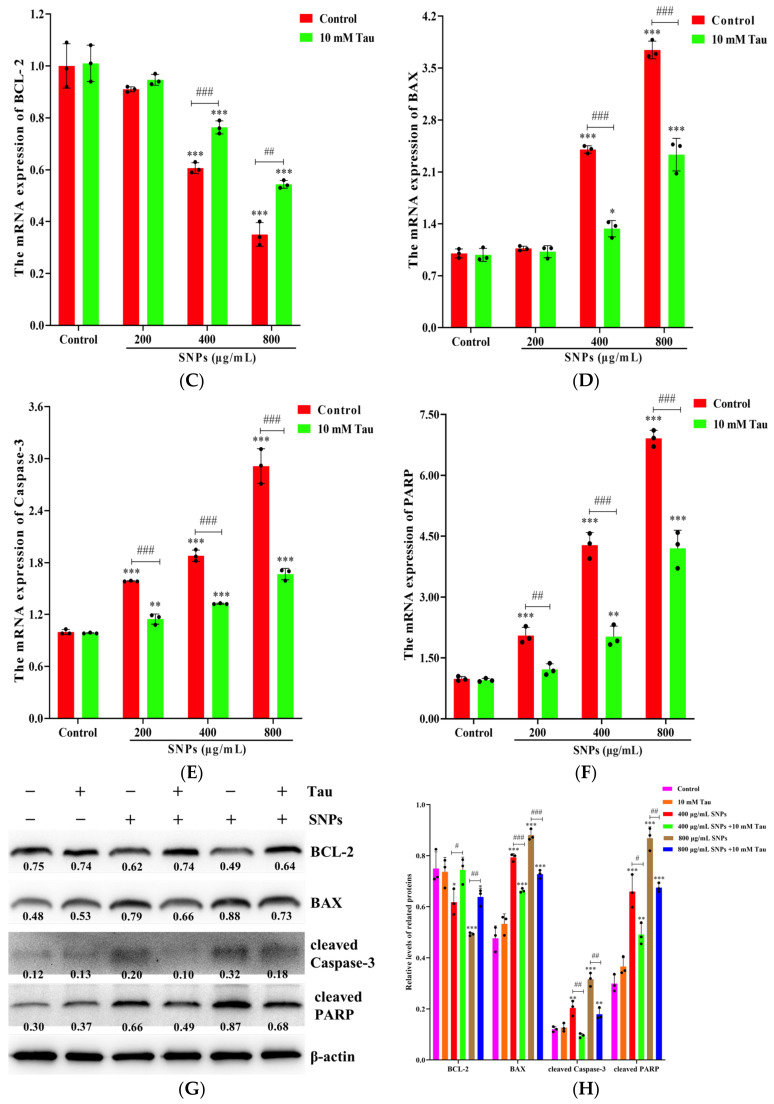
Tau inhibited SNP-induced cell apoptosis in porcine ovarian granulosa cells. Ovarian granulosa cells were exposed to SNPs in the absence or presence of 10 mM Tau for 48 h: (**A**) The apoptotic rate was determined by FCM. Q1-UL quadrant represents cell death caused by mechanical damage or necrotic cells, Q1-UR quadrant represents late apoptotic cells, Q1-LL quadrant represents the normal cells, and Q1-LR quadrant represents early apoptotic cells. (**B**) Quantification of the apoptotic rate. (**B**) Quantification of the apoptotic rate. (**C**–**F**) Quantitative analysis of the mRNA levels for BCL-2 (**C**), BAX (**D**), Caspase-3 (**E**), and PARP (F) by qPCR. (**G**) Detection of BCL-2, BAX, cleaved Caspase-3, and cleaved PARP expressions by Western Blot. (**H**) Quantitative analysis of the band intensity for BCL-2, BAX, cleaved Caspase-3, and cleaved PARP. * *p* < 0.05, ** *p* < 0.01, and *** *p* < 0.001 vs. Control. ^#^ *p* < 0.05, ^##^ *p* < 0.01, and ^###^ *p* < 0.001 vs. SNP-exposed group.

To examine SNP-induced cell apoptosis, FACS results showed that Tau inhibited SNP-induced cell apoptosis (Figure 8A,B). The apoptotic rates in the Tau + 400 μg/mL SNP-exposed group ((11.87 ± 0.10)%) and the Tau + 800 μg/mL SNP-exposed group ((12.05 ± 0.80)%) were lower than those in the 400 μg/mL SNP-exposed group ((14.47 ± 0.20)%) and the 800 μg/mL SNP-exposed group ((17.67 ± 0.23)%) for 48 h, respectively (Figure 8A,B). qPCR results showed that Tau decreased mRNA levels of the cell apoptosis-related genes including BAX, Caspase-3, and PARP while increasing BCL-2 compared to the SNP-exposed groups (Figure 8C–F). Furthermore, Western Blot results showed that Tau decreased the protein expressions of cleaved PARP, cleaved Caspase-3, and BAX, while increasing BCL-2 expression compared to the SNP-exposed groups (Figure 8G,H).

## 4. Discussion

SNPs have garnered significant attention in the research and development of feed additives [1], veterinary drugs [2], and animal vaccines [3,4,5] due to their special physicochemical properties. However, the toxic effects of SNPs are also largely influenced by their physicochemical properties, such as small size and large surface area. In the current study, we assessed SNP-induced cytotoxicity and the protective roles of exogenous Tau administration in porcine ovarian granulosa cells. Our findings demonstrated that SNPs activated oxidative stress through excessive ROS production, leading to inflammation response and cell apoptosis in porcine ovarian granulosa cells. Tau administration significantly decreased SNP-induced oxidative injury and alleviated SNP-induced inflammatory response and cell apoptosis.

Due to their small particle size, SNPs are able to enter the blood circulation through the blood vessel wall and are transported to various organs in the body and accumulate in target or non-target organs, resulting in acute and chronic nanotoxicity [32]. SNPs can penetrate the blood–testis barrier, accumulate in the testes, reduce sperm count and motility, and induce cell apoptosis of testicular interstitial cells, resulting in disturbance of testosterone secretion [14,33]. SNPs also penetrate the blood–placental barrier entering the fetus, causing metabolic disorders both in the mother and fetus [34]. The blood vessels of the ovaries have a unique interlocking network structure in female animals, and lack the protective barriers found in the blood–testis and blood–placental barrier. This unique structure encourages SNPs in the blood to accumulate more easily in the ovaries, allowing them to reach areas close to the oocyte. Previous studies have reported that SNPs significantly cause an increase in ovarian cortical interstitial tissue, thicken cortical and medullary blood vessels, reduce ovarian weight and the number of mature follicles, and increase the number of atretic follicles [19,21].

SNPs reach cells and enter the cytoplasm or the nucleus through diffusion or endocytosis and are degraded via early endosomes–late endosomes/endolysosome vesicle pathway [35]. Similarly, our results showed that SNP particles also entered ovarian granulosa cells and were mainly enclosed in the endosomes in porcine ovarian granulosa cells. Furthermore, to assess the effect of SNPs on cell membrane structure in the process of entering the cell, LDH levels showed a significant difference after SNP exposures. These results indicated that SNPs enter the cytoplasm of porcine ovarian granulosa cells through diffusion or endocytosis leading to damage of cell structure.

Due to their large specific surface area, SNPs exhibit significant redox reaction activity, inducing excessive ROS production. ROS-mediated oxidative stress leads to cytotoxicity, including cell apoptosis, inflammation response, and autophagy. In terms of the mechanism of ovarian toxicity induced by SNPs, Liu et al. found that SNPs primarily cause DNA damage, leading to the activation of the mitochondrial-mediated apoptotic pathway through oxidative stress in mouse ovarian granulosa cells in vivo [18]. Similarly, our recent studies also demonstrated that SNPs cause autophagy dysfunction, resulting in cell apoptosis in mouse ovarian granulosa cells, while *N*-acetylcysteine (NAC), an antioxidant, significantly ameliorated autophagy dysfunction and decreased cell apoptosis [21]. This study showed that SNPs increased ROS accumulation in porcine ovarian granulosa cells. Antioxidants, such as CAT and SOD, play essential roles in maintaining the cellular redox homeostasis by eliminating excess ROS. SNPs inhibited CAT and SOD enzyme activities, impairing their antioxidant roles. The above results indicated that SNPs not only produced excessive ROS but also impaired antioxidant defense systems in porcine ovarian granulosa cells.

Excessive ROS production triggers the activation of inflammatory response and further induces cell death. To elucidate the effect of SNPs on the cytotoxicity in porcine ovarian granulosa cells, inflammation response and cell apoptosis were examined. Clearly, SNPs activated the inflammatory response and induced cell apoptosis in porcine ovarian granulosa cells. Due to the excessive release of ROS, SNPs led to the induction of pro-inflammatory cytokine transcriptions and secretions, such as IFN-α, IFN-β, IL-1β, and IL-6, initiating a strong inflammatory response. However, the SNP-driven transcription and translation effects on pro-inflammatory cytokines remain unclear. Previous studies found that SNP-induced HIF-1α-mediated activation of NOD-like receptor thermal protein domain associated protein 3 (NLRP3) inflammasome leads to the release of pro-inflammatory cytokines, mediated by excessive ROS [36]. Since SNPs cannot be degraded in the cells, the persistent induction of ROS production ultimately results in cell death [37].

Mitochondrial damage plays a crucial role in SNP-induced cell death, with cell apoptosis being the most common type [38]. Previous studies have demonstrated that mitochondria are highly sensitive to nanomaterial exposure and have little tolerance for damage due to the self-generation of ROS [39]. SNPs cause structural abnormalities in mitochondria, leading to dysfunction, including increased mitochondrial permeability, decreased mitochondrial membrane potential (MMP), and imbalanced calcium homeostasis. Our preliminary study found that SNPs mediate calcium mobilization, leading to activation of the mitochondrial apoptotic pathway and cell apoptosis in mouse ovarian granulosa cells [19]. SNPs decrease BCL-2 expression, increase BAX expression and mitochondrial permeability, elevate cytochrome C (Cyt C) release from the mitochondria to the cytoplasm, and cleave Caspase-3 and PARP to trigger mitochondria-dependent apoptosis. Our findings showed that SNPs caused mitochondrial dysfunction by decreasing BCL-2, and increasing BAX, cleaved Caspase-3, and cleaved PARP, resulting in cell apoptosis in porcine ovarian granulosa cells.

To alleviate SNP-induced toxicity, one of the main strategies is to scavenge excess ROS. Tau, an endogenous amino acid with antioxidant properties, has been reported to mitigate reproductive toxicity. However, the effect of Tau on nanoparticle toxicity is not documented well [40,41]. Previous studies found that Tau protects against silver nanoparticle-induced neurotoxicity [40] and reproductive toxicity [41] by the attenuation of oxidative stress-mediated inflammatory response and caspase-3 activity in rats in vivo. In the current study, we investigated the addition of Tau to attenuate oxidative stress and reduce SNP-induced cytotoxicity in porcine ovarian granulosa cells. According to previous reports, 5–20 mM taurine promotes estrogen synthesis in mice ovarian granulosa cells [42], improves blastocyst yield from poor-quality feline oocytes in vitro [43], and supports 2–4-cell human embryos development to the blastocyst stage [44]. Our findings revealed that exogenous Tau supplementation significantly decreased ROS accumulation. Although Tau does not directly scavenge excess ROS, it functions as an indirect antioxidant by reducing ROS accumulation through the mitochondrial electron transport chain (ETC) complex I [45]. SNP-induced oxidative stress inhibited the activities of CAT and SOD enzymes while Tau elevated the activity of these antioxidant enzymes. These results are consistent with previous findings [46,47], which show an increase in the levels of the antioxidant defense system, primarily by protecting the antioxidant enzymes against oxidative stress damage. However, the mechanism by which Tau decreased ROS accumulation needs further exploration after SNP exposure in porcine ovarian granulosa cells.

Tau exhibits anti-inflammatory properties by blocking oxidative stress and inflammatory responses. Previous studies have shown that Tau reduces the secretion of pro-inflammatory cytokines, such as TNF-α, NF-κB, and IL-1β, in bleomycin- [48], endotoxin- [49], and cigarette smoke- [50] induced acute lung injury. Tau also reduces TNF-α and IL-6, alleviating the severity of brain injuries [51]. Moreover, Tau protects against cisplatin-induced gonadotoxicity by reducing inflammation [52]. The current study found that exogenous Tau supplementation reduced INF-α, INF-β, IL-1β, and IL-6, showing anti-inflammatory effects. Toll-like receptors (TLRs) are a class of pattern recognition receptors in innate immunity to eliminate pathogenic invasion. Tau and its metabolites, such as taurine-bromamine (TauBr) and taurine-chloramine (TauCl), exert anti-inflammatory properties through the TLR/Myeloid differentiation primary response 88/Nuclear factor kappa-light-chain-enhancer of activated β cells (TLR/MYD88/NF-κβ) signal transduction pathway [53]. SNPs increase the release of IL-1β via the activation of the TLR/MYD88/NF-κβ signaling pathway in dendritic cells [54]. SNPs also induce TNF-α, IL-1β, and IL-6 through the activation of the HMGB1/TLR4/MYD88/NF-κB signaling pathway in HUVEC cells [36]. Additionally, SNPs increase the release of IL-1β and TNF-α via TLR/MYD88/NF-κB cascade in the oxidative injury of the lung, and arctiin and arctigenin suppress the activation of this pathway to reduce inflammation [55]. So, we speculated that Tau may exert its anti-inflammatory effects by suppressing the SNP-activated TLR4/MYD88/NF-κB signaling pathway in the porcine ovarian granulosa cells, which needs further investigation.

SNPs can induce cell apoptosis via mitochondrial damage, while Tau protects against mitochondrial dysfunction to inhibit cell apoptosis. Tau maintains intracellular calcium homeostasis by preventing an increase in the mitochondrial calcium level [56]. Additionally, Tau inhibits cleavage of Caspase-9 and Caspase-3 by suppressing Apaf-1/Caspase-9 apoptosome formation during ischemia [57]. Tau provides effective protection against deoxynivalenol-induced liver injury by restoring normal mitochondrial function [58]. Tau attenuates Caspase-3 activation, protecting against silver nanoparticle-induced neurotoxicity [40]. The current study showed that Tau inhibited SNP-induced cell apoptosis by increasing BCL-2, and decreasing BAX, cleaved Caspase-3, and cleaved PARP in the porcine ovarian granulosa cells. The above findings indicate that Tau inhibits mitochondria-mediated apoptosis after SNP exposures in porcine ovarian granulosa cells.

## 5. Conclusions

This study reveals the effects of SNP-induced ovarian toxicity. Tau ameliorates SNP-induced cell apoptosis by reducing oxidative stress, inflammatory response, and cell apoptosis in porcine ovarian granulosa cells. Tau administration may be an effective strategy to alleviate SNP-induced toxicity. Additionally, Tau-conjugated SNPs could have promising applications in animal husbandry and the veterinary industry.

## Figures and Tables

**Table 1:** Sequences of the primers of target genes for qPCR.

Gene	Gene Bank No.	Forward (5′-3′)	Reverse (5′-3′)	Product (bp)
*CAT*	NM_214301.2	CCAGCCAGTGACCAGATGAAG	ACACCTTCGCCTTCGAGAAT	280
*SOD1*	NM_001190422.1	TGACTGCTGGCAAAGATGGT	TTTCCACCTCTGCCCAAGTC	133
*IFN-α*	JQ839262.1	AGGAGAATCTCTCCCTTCTCC	GAGCCCTCTGTGCTGAAGAG	155
*IFN-β*	GQ415073.1	ACCAACAAAGGAGCAGCAA	TCAGGGACCTCAAAGTTCATC	103
*IL-1β*	NM_214055.1	ACCTGGACCTTGGTTCTCTG	CATCTGCCTGATGCTCTTGT	83
*IL-6*	AF518322.1	CTGGCAGAAAACAACCTGAACC	TGATTCTCATCAAGCAGGTCTCC	94
*BCL-2*	XM_021099593.1	TCCAGAACCTCCTTGGTCCT	AACTACAGCGAGGTGCTTCC	187
*BAX*	XM_003127290.5	GCTTCAGGGTTTCATCCAGGATCG	ACTCGCTCAACTTCTTGGTAGATGC	107
*Caspase-3*	NM_214131.1	AGAATTGGACTGTGGGATTGAGACG	GCCAGGAATAGTAACCAGGTGCTG	122
*PARP*	XM_001927325.2	GATACTGGACTGCTGCTC	ACTCCCATTCAAGGTGAT	162
*GAPDH*	NM_001206359.1	GTCGGTTGTGGATCTGACCT	TTGACGAAGTGGTCGTTGAG	207

## Data Availability

The data that support the findings of this study are available upon request.

## Supplementary Materials

The following supporting information can be downloaded at: https://www.mdpi.com/article/10.3390/ani14202959/s1, Figure S1: Detection of pure primary porcine ovarian granulosa cells; Figure S2: The original Western blot images.
