# Supplementary material for: Taurine Protects against Silica Nanoparticle-Induced Apoptosis and Inflammatory Response via Inhibition of Oxidative Stress in Porcine Ovarian Granulosa Cells
Source: Animals (Basel). 2024 Oct 14;14(20):2959. doi: 10.3390/ani14202959 (PMC11506286; doi:10.3390/ani14202959)
Supplement: Supplementary file 1 [file animals-14-02959-s001.zip › animals-3189668-supplementary_1.pdf]

Supplementary material

Taurine protects against silica nanoparticle-induced apoptosis and inflammatory response via inhibition of oxidative stress in porcine ovarian granulosa cells

Fenglei Chen [1,2,3*], Jiarong Sun [1], Rongrong Ye [1], Tuba Latif Virk [1], Qi Liu [1,2,3], Yuguo Yuan [1,2,3] and Xianyu Xu [1,2,3*]

[1] College of Veterinary Medicine, Yangzhou University, Yangzhou 225009, China; flchen@yzu.edu.cn (F.L. Chen); 15996825403@163.com (J.R. Sun); yrr971106@163.com (R.R. Ye); xeroxhunt@gmail.com (T. L. Virk); qiliu@yzu.edu.cn (Q. Liu); yyg9776430@163.com (Y.G. Yuan);xuxianyu@yzu.edu.cn (X.Y. Xu).

[2] Jiangsu Co-innovation Center for Prevention and Control of Important Animal Infectious Diseases and Zoonoses, Yangzhou 225009, China; flchen@yzu.edu.cn (F.L. Chen); qiliu@yzu.edu.cn (Q. Liu); yyg9776430@163.com (Y.G. Yuan);xuxianyu@yzu.edu.cn (X.Y. Xu).

[3] Joint International Research Laboratory of Agriculture and Agri-Product Safety of the Ministry of Education of China, Yangzhou University, Yangzhou 225009, China; flchen@yzu.edu.cn (F.L. Chen); qiliu@yzu.edu.cn (Q. Liu); yyg9776430@163.com (Y.G. Yuan);xuxianyu@yzu.edu.cn (X.Y. Xu).

* Correspondence: flchen@yzu.edu.cn (F.L. Chen); xuxianyu@yzu.edu.cn (X.Y. Xu); Tel.: +86-514-8797-9030; Fax: +86-514-8797-2218.

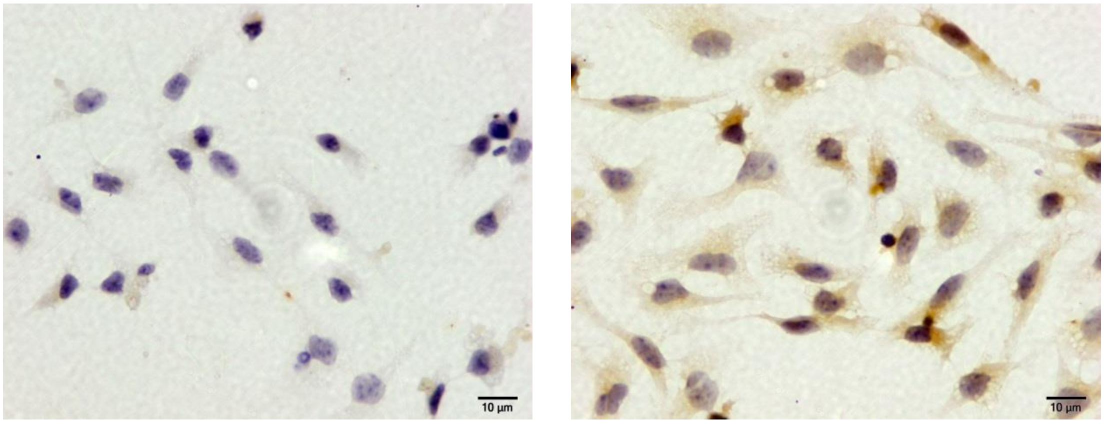

(A) (B)

Figure S1. Detection of pure primary porcine ovarian granulosa cells. (A) Representative image of the negative control by immunohistochemistry. (B) Representative image of FSHR staining by immunohistochemistry. Scale bar, 10 μm.
