# Supplementary material for: Taurine Protects against Silica Nanoparticle-Induced Apoptosis and Inflammatory Response via Inhibition of Oxidative Stress in Porcine Ovarian Granulosa Cells
Source: Animals (Basel). 2024 Oct 14;14(20):2959. doi: 10.3390/ani14202959 (PMC11506286; doi:10.3390/ani14202959)
Supplement: Supplementary file 1 [file animals-14-02959-s001.zip › animals-3189668-supplementary_2.pdf]

Figure S2

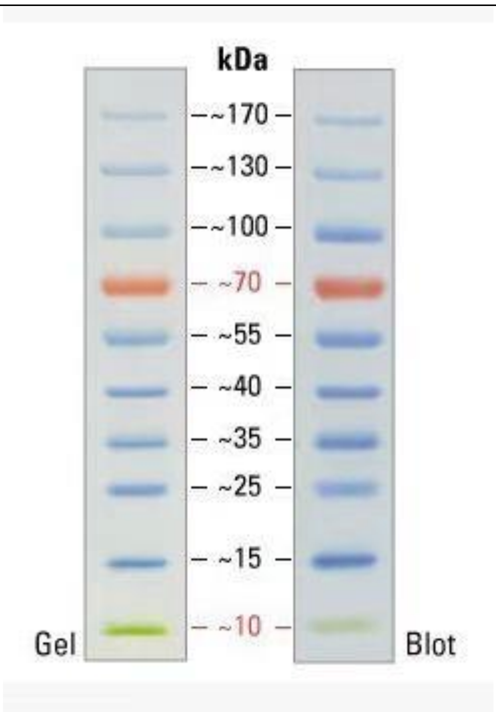	Markers
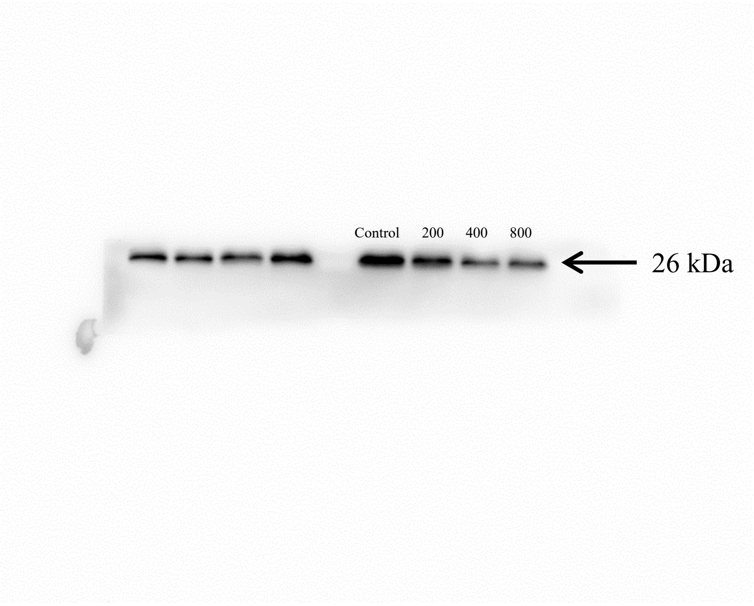	BCL-2

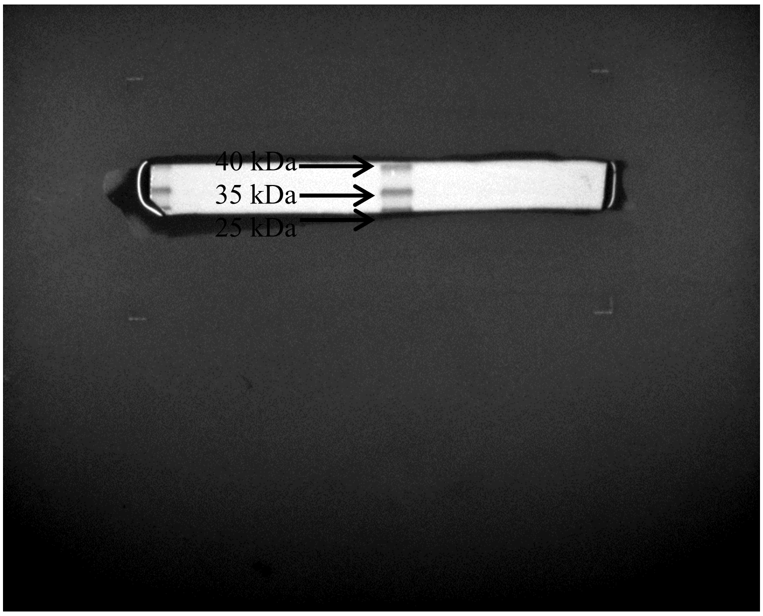	
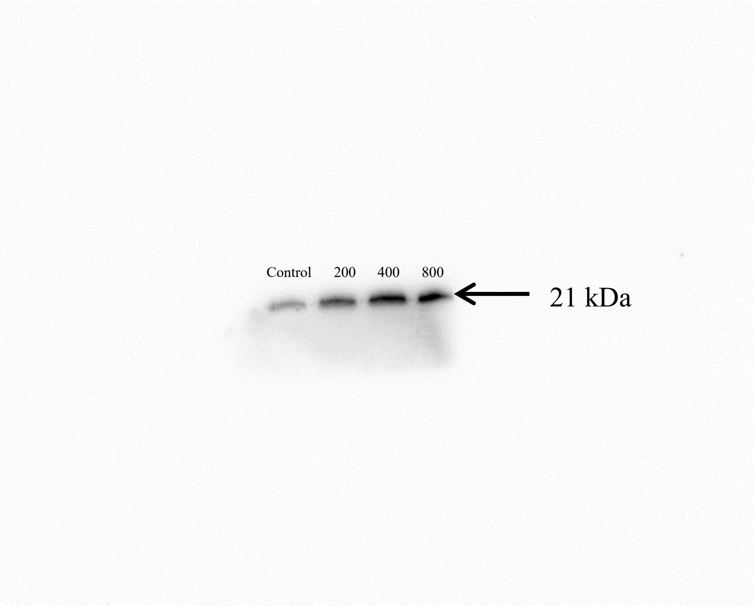	BAX

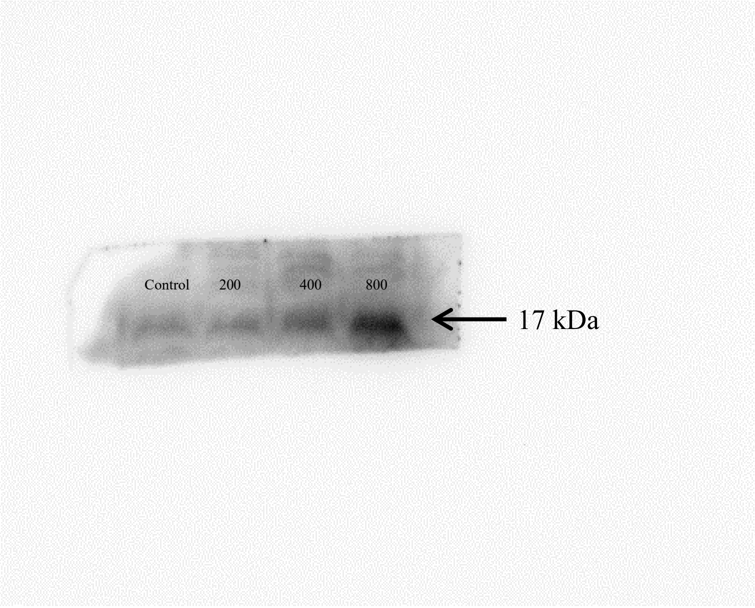	cleaved Caspase-3
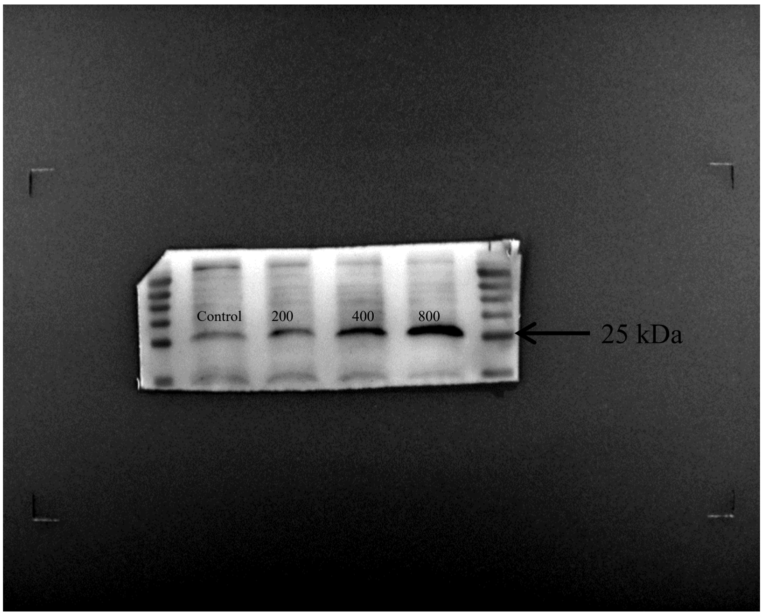	cleaved PARP

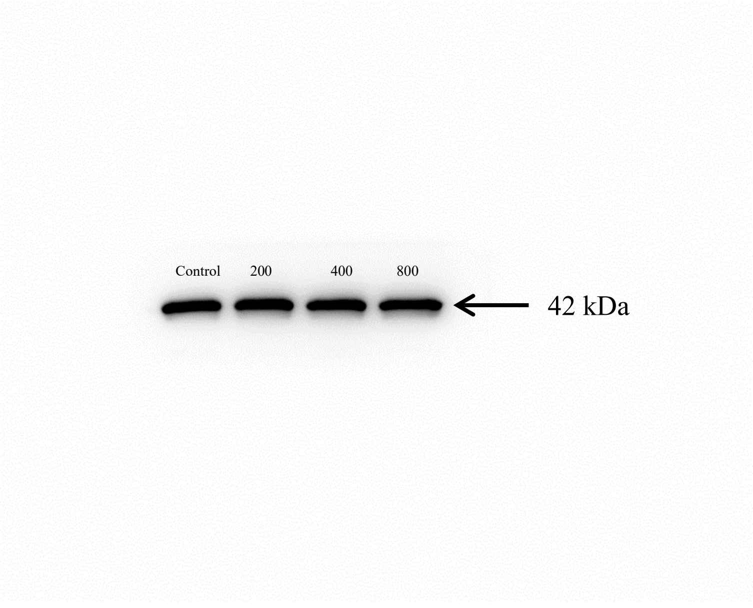	**β-actin**

Figure 8F

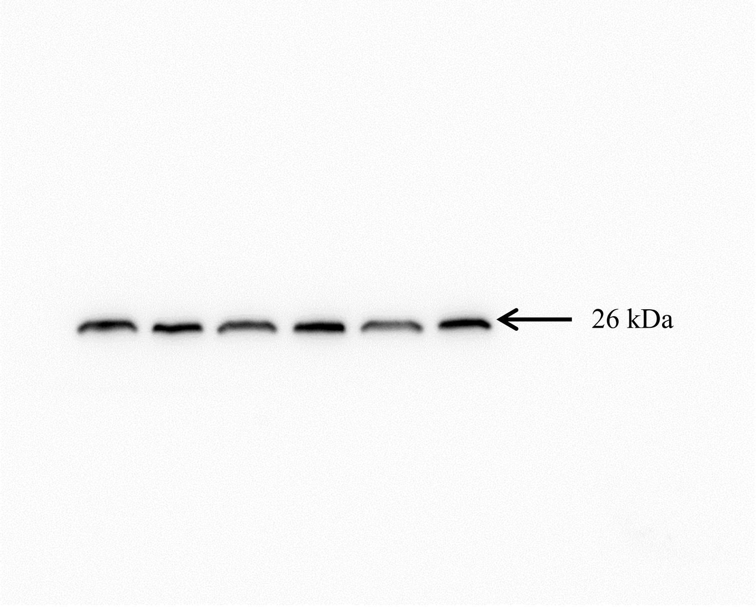	BCL-2

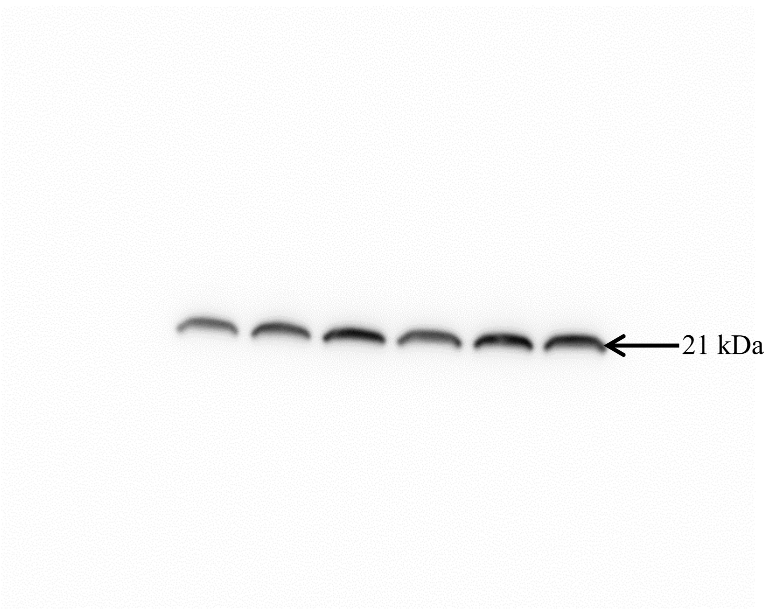	BAX
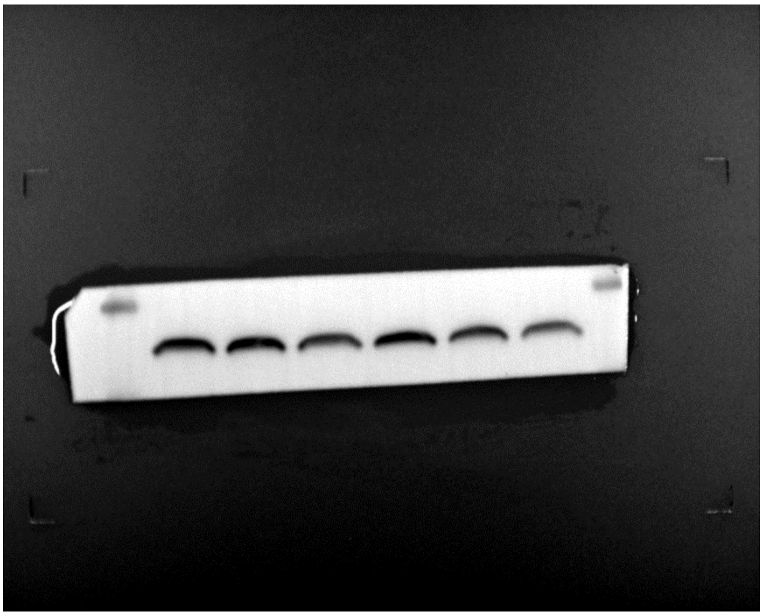	

<table>
<tr><td>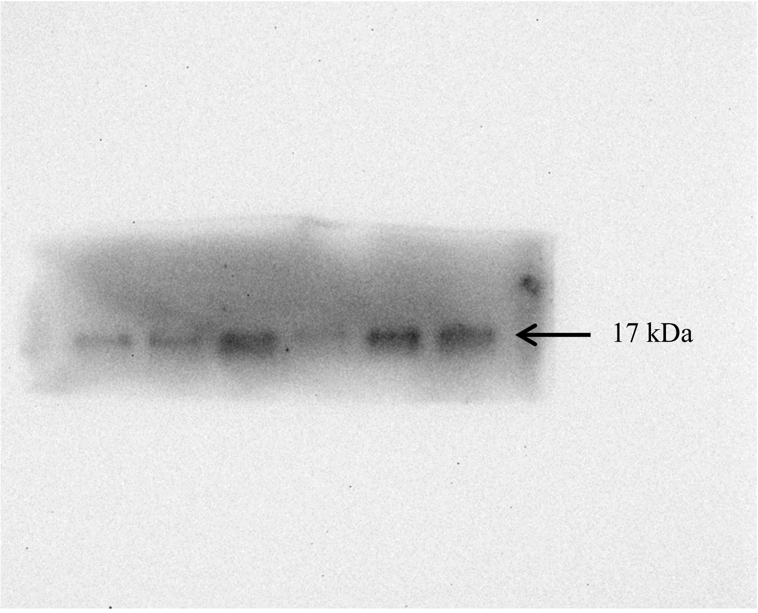
</td><td rowspan="2">Cleaved Caspase-3</td></tr>
<tr><td>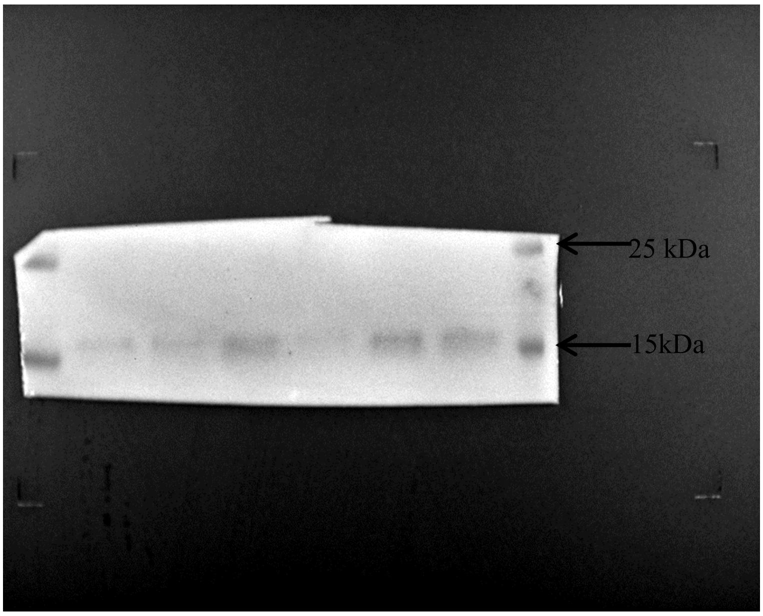
</td></tr>
</table>

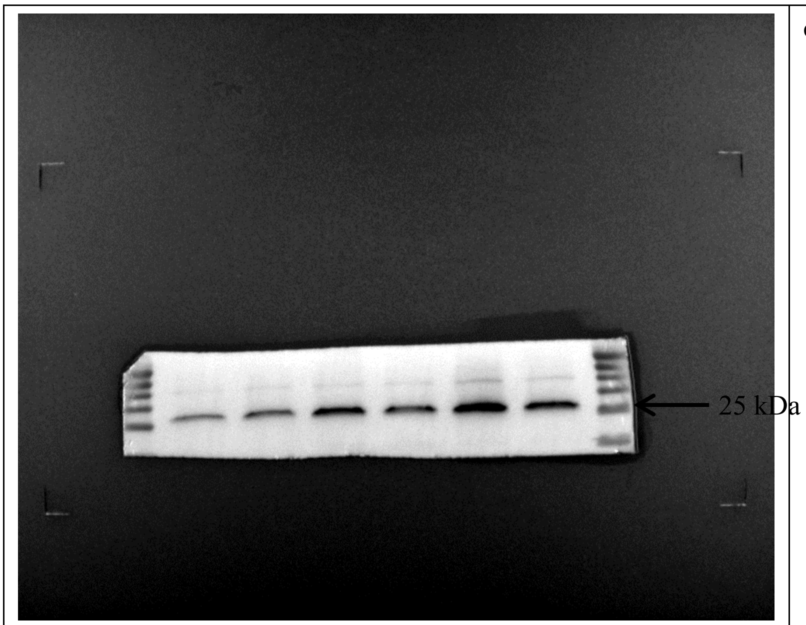	cleaved PARP
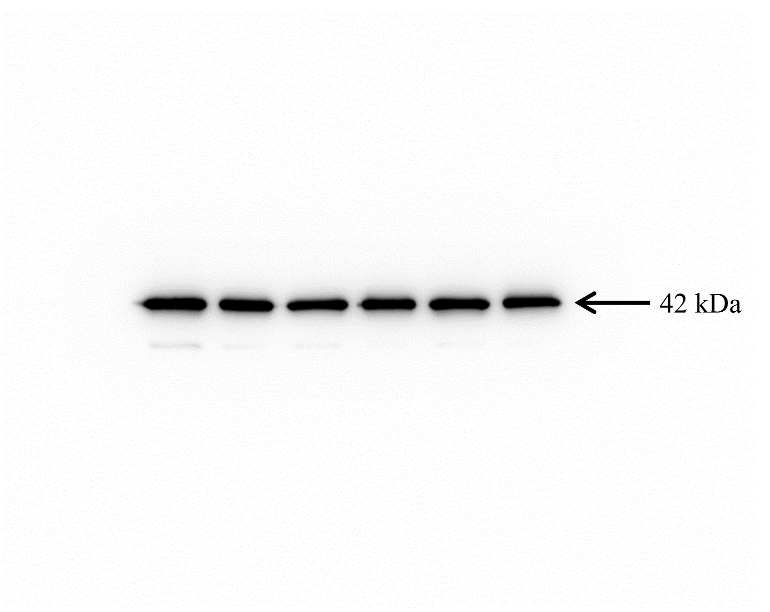	β-actin
